# Prevalence and genotype distribution of cervical HPV among women living with and without HIV in selected health facilities in Limpopo province, South Africa

**DOI:** 10.4102/sajid.v40i1.747

**Published:** 2025-12-17

**Authors:** Rixongile R. Rikhotso, Emma M. Mitchell, Pascal O. Bessong

**Affiliations:** 1 Department of Biochemistry and Microbiology, Faculty of Science, Engineering and Agriculture, University of Venda, Thohoyandou, South Africa; 2 Department of Nursing Research, School of Nursing, University of Virginia, Charlottesville, United States; 3 Center for Global Health Equity, University of Virginia, Charlottesville, United States; 4 School of Health Sciences, University of KwaZulu-Natal, Durban, South Africa; 5 South African Medical Research Council-University of Venda Antimicrobial Resistance and Global Health Research Unit, Faculty of Science, Engineering and Agriculture, University of Venda, Thohoyandou, South Africa

**Keywords:** human papillomavirus, prevalence, genotypes, HIV, next generation sequencing, South Africa

## Abstract

**Background:**

High-risk alpha human papillomaviruses (HPVs) are associated with cervical cancer (CC).

**Objectives:**

This study investigated the prevalence and genotype distribution of HPV among women living with and without HIV in Limpopo province, South Africa.

**Method:**

The prevalence of HPV was determined in 450 participants who self-reported to be living with HIV or not. Total DNA was extracted from cervical specimens and amplified through a double-nested polymerase chain reaction (PCR) approach targeting a fragment of the *L1* gene. A product from any of the nested PCRs was considered positive for the presence of HPV DNA. The first nested PCR products (~450 base pairs [bp]) were sequenced on an Illumina MiniSeq. Sequence reads of acceptable quality were analysed for viral genotypes.

**Results:**

Human papillomavirus was detected in 32.7% of the study participants and was significantly higher at 52.21% (*p* = 0.00) among women living with HIV (WLWH) as compared to those not living with HIV. Overall, high-risk (hr) HPV 45 was the predominant genotype (16.7%). However, low-risk (lr) HPV 81 (18.8%) and hr-HPV 56 (25.0%) were more common among women living with and without HIV, respectively. Multiple infections and hr-HPV genotypes were more common among WLWH.

**Conclusion:**

A relatively high prevalence of HPV was detected in the cervical specimens from the study population. Women living with HIV were the most infected group. The data suggest that WLWH should be prioritised for HPV screening and vaccination.

**Contribution:**

This study contributes to the knowledge of HPV in Limpopo province, a region with scarce data on HPV epidemiology.

## Introduction

Human papillomaviruses (HPVs) are heterogeneous double-stranded DNA viruses from the *Papillomaviridae* family. These viruses are phylogenetically grouped into five genera: *Mu-, Nu-, Gamma-, Beta- and Alphapapillomavirus*.^1^ Furthermore, based on their carcinogenic properties, HPVs are classified as high-risk (hr) and low-risk (lr) genotypes.^2^ Over 200 HPV genotypes have been identified,^1^ with genital infections caused by unique pathogenic HPV genotypes of the *Alphapapillomavirus* genus.^3^ The HPV genome size is about 8 kilobases, with the *L1*, a conserved gene encoding the major capsid protein.^4^ Since the discovery of genital HPV in the 1970s, these diverse groups of viruses have been identified worldwide, including South Africa.^5,6,7^ Mostly, mucosal alpha HPVs are transmitted through sexual contact^8^ and are a major cause of cervical cancer (CC).^9^

Worldwide, over 99% of CC cases are caused by persistent hr-HPV infection.^10^ Globally, CC is the fourth leading cause of cancer-related deaths among women.^11^ In addition, sub-Saharan Africa reports the highest CC rates^11^ with high HIV prevalence being the contributing factor.^12^ In South Africa, CC ranks second to breast cancer,^13^ with an estimated 10 532 new CC cases in 2022, which resulted in approximately 5976 deaths.^14^ By global comparisons, South Africa has the highest rates of HIV prevalence, with approximately 7.8 million individuals living with HIV.^15^ An association between HIV and specific cancers has been established.^16^ The high prevalence of HIV among South African women renders HPV-HIV co-infection a significant public health concern, one that calls for intensified surveillance and tailored interventions.

Human papillomavirus prevalence varies among countries and by lifestyle and age.^17^ Globally, the estimated HPV prevalence in women with normal cervical cytology is 11.7%, with the highest prevalence again reported in sub-Saharan Africa (24.0%).^18^ South African studies report varying viral prevalences, with up to 96.6% reported among women diagnosed with cervical intraepithelial neoplasia III.^19^ Other studies show higher HPV prevalence among women living with HIV (WLWH) compared to those living without^20,21^; meanwhile, others report no difference.^6^ Globally, lr-HPV 6, lr-HPV 11, hr-HPV 16 and hr-HPV 18 are generally the predominant genotypes.^22,23^ However, studies show variations. In Tanzania, hr-HPV 16 and hr-HPV 58 were predominant hr-HPV genotypes, while hr-HPV 53 was also prevalent among pregnant women.^24^ In South Africa, hr-HPV 16, hr-HPV 52 and hr-HPV 53 were among the most detected.^25^ A recent study from South Africa detected lr-HPV 42 as the most common lr-HPV genotype.^26^ Moreover, a sentinel surveillance study reported hr-HPV 16, followed by lr-HPV 61, lr-HPV 81 and lr-HPV 83 as the predominant genotypes in South Africa.^27^ Therefore, the distribution of HPV genotypes is heterogeneous across populations. Because of the highly distinct genotypes of HPV, it is important to study the virus in different geographic locations, people, and persons living with HIV to inform the selection of genes for vaccine improvements and potential vaccine trial sites.

The HPV genotyping kits primarily utilise polymerase chain reaction (PCR) hybridisation-based assays, which have gained popularity in South Africa.^28^ These tests, although useful, are limited to detecting only known genotypes. Nevertheless, PCR remains an important method for HPV DNA detection because it enables amplification of targeted regions, which can be studied further. Next-generation sequencing (NGS) may lead to the detection of viral genotypes existing as a minority population,^29^ and could further detect a wider spectrum of HPV genotypes than other methods.^30^ Cervical HPV DNA genotyping is a fundamental method to identify clinically relevant genotypes. In this study, the *L1* conserved region of HPV was amplified using a double-nested PCR approach, followed by NGS to enhance sensitivity and reliability for HPV detection.^31^ Investigating HPV prevalence and genotype distribution in the Limpopo province is crucial as there is a paucity of such data using the PCR-NGS strategy.

## Research methods and design

### Study design, participant recruitment and sample size

This analytical cross-sectional study comprised 450 women aged ≥18 years who self-reported living with or without HIV, between March 2017 and February 2019. These participants included those visiting health facilities for HIV treatment and others as part of routine healthcare services. Furthermore, the participants were recruited from four selected public health facilities located in the Vhembe (Thohoyandou Health Centre and Donald Frazer Hospital) and Capricorn (Rethabile Community Health Centre and Seshego Clinic) districts in Limpopo province. Notably, none of the participants were vaccinated against HPV.

### Inclusion and exclusion criteria

Eligible participants were women aged ≥ 18 years, residing in Limpopo province, willing to provide informed consent and without a history of a CC diagnosis. Excluded criteria included pregnant women, a history of hysterectomy, undergoing treatment for a sexually transmitted infection (STI), and current vaginal bleeding or discharge.

### Data collection

A structured questionnaire was used to collect participants’ demographic information (age, income level, marital status, age at first sexual debut, type of sexual partner, and educational level) and clinical data (HIV and HPV vaccination status). Two independent individuals verified the collected information for accuracy and consistency after the data were captured.

### Specimen collection and DNA extraction

Cervical specimens were collected using a blue shaft brush by qualified study nurses. This was performed using the Aptima Cervical Collection and Transport Kit as per the manufacturer’s protocol (Hologic, San Diego, CA, United States). Total DNA was extracted from the cervical specimens using a QIAamp DNA mini kit (Qiagen, Hilden, Germany), following the manufacturer’s instructions. However, in the DNA elution step, 100 µL of eluent was used instead of 200 µL and was stored at −20 °C.

### Detection of human papillomavirus DNA by double-nested polymerase chain reaction

The PCR cycling conditions were adapted from Tawe et al.^32^ The DNA extract was first amplified through PCR using SB01/02 primers^33^ to amplify ~495 base pairs [bp] fragment of the *L1* gene. A volume of 10 µL of cervical DNA extract was added into the PCR reaction mixture, which contained 3.5 mM of MgCl_2,_ 0.4 µM of each reverse and forward primer, 0.08 U/µL of FastStart Taq DNA Polymerase, 0.2 mM dNTPs, 2X PCR buffer and 3.6 µL PCR-grade water in a final reaction volume of 25 µL. The first nested reaction, using MY09/11 primers,^34^ was expected to yield an HPV DNA product of ~450 bp. Briefly, the reaction comprised: 5 µL DNA template, 3.5 mM MgCl_2_, 0.4 µM of each reverse and forward primer, 0.2 mM dNTPs, 0.08 U/µL of FastStart Taq DNA polymerase, 2X PCR buffer and 8.6 µL PCR water to achieve a final volume of 25 µL. Negative reactions from the first nested reaction were subjected to another nested reaction using GP5/6 primers^35^ and amplified an expected HPV DNA product of ~150 bp. This reaction constituted the same PCR reaction mixture as the first nested reaction without any modifications. The resultant PCR products were ascertained by 2.5% gel electrophoresis for expected band size verification. Notably, to ensure successful amplification and the HPV protocol reliability for all the reactions, a previously confirmed HPV specimen was used as a positive control. The prevalence of HPV DNA in the study population was determined by adding the number of DNA positives in the first and second nested reactions and then expressing it as a percentage.

### Next-generation sequencing with the Illumina MiniSeq platform for human papillomavirus genotyping

Human papillomavirus genotypes were determined using the 450 bp PCR products. Briefly, detected HPV DNA was cleaned using AMPure XP beads (Beckman Coulter, California, United States). DNA libraries were prepared using the Nextera XT DNA library preparation kit (Illumina San Diego, California, United States) and were sequenced using the Illumina MiniSeq platform according to the manufacturer’s instructions. Sequence reads were quality-checked before analysis, using the FastQC tool. Briefly, the generated FastQ sequences with a Phred score of ≥ 30 were identified and used for further downstream applications.

### Human papillomavirus genotyping

Quality-assured FastQ sequences were imported into Geneious Prime software (version 2023.0.1, Dotmatics, Boston, United States). The HPV reference genomes were downloaded from the Papillomavirus Episteme (PaVE)^1^ database, accessed on 30 May 2022. Study sequences were mapped to various HPV genotypes and the identified genotypes were examined for their distribution in the study population.

### Data analysis

Statistical analysis was performed using R software version 4.4.1 (The R Foundation, Vienna, Austria). Most of the data were categorical. Data were double-checked for errors and consistency prior to analysis. When calculating the data as proportions of the total sample, undisclosed data were excluded from the denominator. A bivariate regression model was employed to identify risk factors for HPV DNA positivity. Variables with a *p*-value of less than 20% in the bivariate analysis were included in the final multivariate model, significant at a *p*-value less than 0.05.

### Ethical considerations

Ethical clearance to conduct this study was obtained from the Human and Clinical Trials Research Ethics Committee of the University of Venda (SMNS/20/MBY/12/1003). Before enrolment and specimen collection, informed consent was obtained from each participant. To protect the privacy and confidentiality of each participant, the study samples were anonymised; sample codes instead of participants’ names were used throughout.

## Results

### Characteristics of study participants

A total of 450 black African study participants were enrolled and screened for HPV DNA. Demographic data were available for 356 participants who were within an age range of 18–84 years (mean 41.51, standard deviation [s.d.] 12.03) and predominantly heterosexual (89.04%; *n* = 317). Moreover, most participants (57.87%, *n* = 206) were living without HIV (Table 1).

**TABLE 1:** Sociodemographic and clinical characteristics of the study participants (*N* = 356).

Characteristics	*n*	%
**Age (years)†**		
18–28	53	14.89
29–38	103	28.93
39–48	115	32.30
49–58	47	13.20
≥ 59	38	10.67
**Level of education**		
No educational background	15	4.21
Primary level (grade 1–7)	44	12.36
Secondary level (grade 8–12)	245	68.82
Post grade 12 studies	39	10.96
**Marital status**		
Single	161	45.22
Married	165	46.35
Divorced	3	0.84
Widow	27	7.58
**Employment status**		
Neither disclosed nor specified	11	3.09
Employed	142	39.89
Unemployed	180	50.56
Pensioner	23	6.46
**Income (South African Rand)**		
Neither disclosed nor specified	219	61.52
Less than R3000	73	20.51
Between R3000 and R10 000	29	8.15
Greater than R10 000	35	9.83
**HIV status**		
Positive	150	42.13
Negative	206	57.87
**Tobacco users**		
Neither disclosed nor specified	2	0.56
Smoker	10	2.81
Non-smoker	344	96.63
**HPV vaccination status**		
Neither disclosed nor specified	24	6.74
Not vaccinated	332	93.26
**Age at first sexual intercourse**		
Neither disclosed nor specified	30	8.43
11–15	41	11.52
16–20	234	65.73
21–25	46	12.92
25+	5	1.40
**Type of sexual partner**		
Neither disclosed nor specified	38	10.67
Female	1	0.28
Male	317	89.04
**Number of sexual partners**		
Neither disclosed nor specified	31	8.71
0 or celibate	12	3.37
1–2	310	87.08
3–4	3	0.84

HPV, human papillomavirus.

† , Age (years): Mean ± standard deviation, 41.51 ± 12.0

### Prevalence of human papillomavirus DNA

The prevalence of HPV DNA detected by any of the nested PCR approaches was 32.7% (*n* = 147/450). Human papillomavirus DNA was detected in more specimens (*n* = 95) by the second nested PCR compared to the first nested reaction (*n* = 52). Demographic data were available for 136 out of 147 participants who tested positive for HPV DNA. Of these, women who were living with HIV had a significantly higher HPV positivity rate (52.21%, *n* = 71), compared to women living without HIV (47.79%, *n* = 65), (prevalence odds ratio [POR]: 1.95, 95% confidence interval [CI]: 1.26–3.02, *p* = 0.00).

### Distribution of human papillomavirus genotypes

**FIGURE 1:**
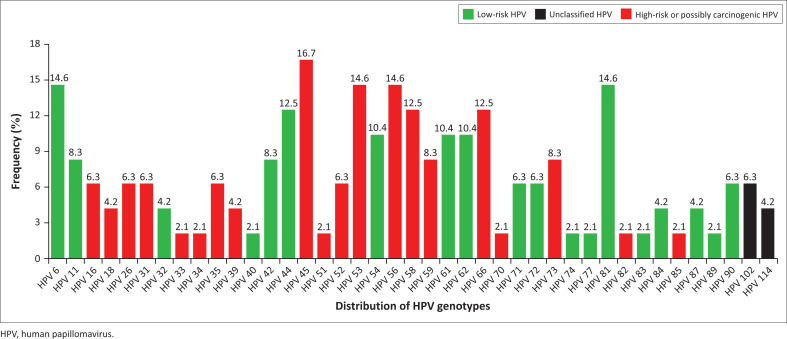
The distribution of 41 human papillomavirus genotypes detected in the study population.

**FIGURE 2:**
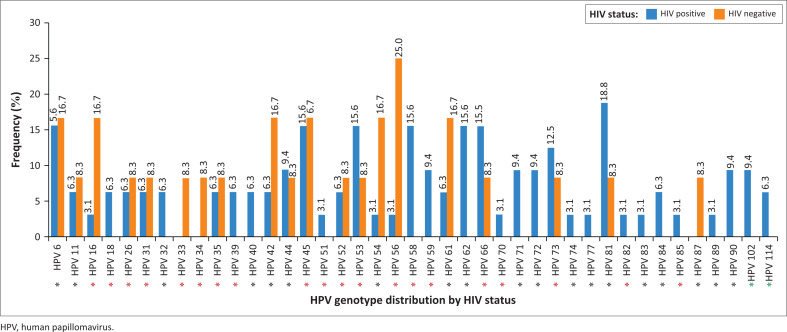
The frequency distribution of human papillomavirus genotypes according to HIV status. Red asterisks denote high-risk and possibly carcinogenic human papillomavirus genotypes, while black and green asterisks indicate low-risk and unclassified genotypes, respectively.

Of the 52 sequenced data of participants, 48 sequences were of acceptable quality. Of these, 32 were living with HIV and 12 without, while the remaining 4 participants had an unknown HIV status. Consequently, a total of 41 HPV genotypes, all from the *Alphapapillomavirus* genus, were identified, as shown in Figure 1. The most commonly detected HPV genotype was hr-HPV 45 (16.7%) irrespective of HIV status. Low-risk HPV 81 (18.8%), followed by lr-HPV 6, hr-HPV 45, hr-HPV 53, hr-HPV 58, lr-HPV 62 and hr-HPV 66 (15.6% each) were predominantly detected among WLWH (Figure 2). In contrast, participants not living with HIV were more frequently infected with lr-HPV 6, hr-HPV 16, lr-HPV 42, hr-HPV 45, HPV lr-54, lr-HPV 61 (16.7% each) and hr-HPV 56 (25.0%) (Figure 2). Women living with HIV were found to have a higher number of HPV genotypes (*n* = 38) compared to women not living with HIV (*n* = 20). In addition, hr-HPV genotypes and multiple infections (59.4%) were higher among WLWH, Figure 2.

### Prevalence and distribution of known carcinogenic human papillomavirus genotypes

Among the 25 known carcinogenic HPV genotypes classified by the International Agency for Research on Cancer,^36^ 20 were detected. In this study, five carcinogenic genotypes, namely: HPV 30, HPV 67, HPV 68, HPV 69 and HPV 97 were not detected. Overall, 79.2% (*n* = 38/48) of participants were infected with at least one carcinogenic HPV genotype. The predominant carcinogenic HPV genotype was hr-HPV 45 (21.1%), followed by hr-HPV 53 and hr-HPV 56 (18.4% each). The majority of WLWH were infected with hr-HPV 45, hr-HPV 53, hr-HPV 58 and hr-HPV 66, with a frequency of 19.2% each. High-risk HPV 16 (25.0%) and hr-HPV 45 (25.0%) were more prevalent among women not living with HIV.

### Prevalence and distribution of human papillomavirus genotypes incorporated in the Gardasil-9 vaccine

**FIGURE 3:**
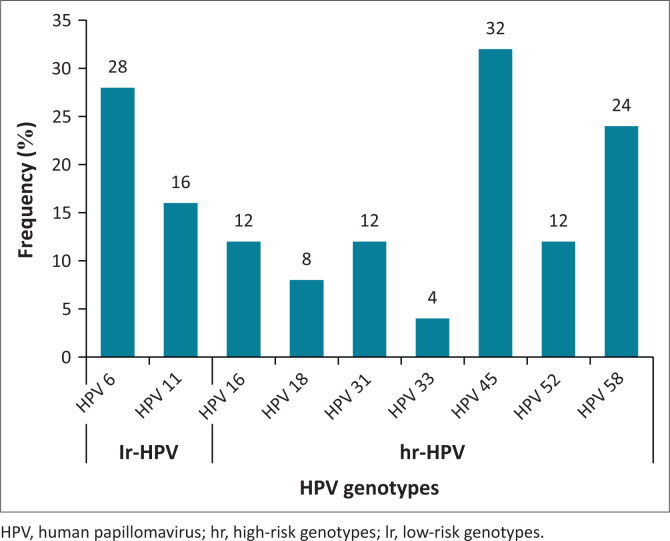
The occurrence of human papillomavirus genotypes covered by the Gardasil-9 human papillomavirus vaccine among study participants.

Genotypes that were observed in this study were compared with the ones incorporated in the most recent vaccine against HPV, Gardasil-9. More than half of the participants (*n* = 25/48, 52.1%) tested positive for at least one of the HPV genotypes incorporated in the Gardasil-9 vaccine, not in use in South Africa, Figure 3. The most frequently detected genotype in the study population was hr-HPV 45 (32.0%). Low-risk HPV 6, hr-HPV 45 and hr-HPV 58 (33.3% each) were more prevalent among WLWH, while those not living with HIV harboured more of lr-HPV 6, hr-HPV 16, hr-HPV 45 and hr-HPV 52, each with a frequency of 33.3%.

### Risk factors associated with human papillomavirus infection

Human immunodeficiency virus status was significantly associated with HPV status. In the bivariate analysis, the risk of being HPV positive among WLWH was approximately twofold as compared to those living without HIV (POR = 1.95, 95% CI: 1.26–3.02, *p* = 0.00). Women aged 49–58 years were also significantly more likely to be HPV positive than those aged 18–28 years (POR = 2.56, 95% CI: 1.14–5.91, *p* = 0.03). Additionally, women with primary level education or less had a significantly lower risk of HPV positivity compared to those with tertiary education (POR = 0.38, 95% CI: 0.16–0.89, *p* = 0.02). Variables such as employment, income level and marital status were not significantly associated with HPV positivity. Even after multivariate adjustment, living with HIV remained a significant risk factor for HPV positivity, with WLWH having approximately twice the risk compared to women without HIV (POR =1.97, 95% CI: 1.24–3.16, *p* = 0.00). Similarly, women aged 49–58 years continued to show a significantly higher risk of being HPV positive compared to those aged 18–28 years (reference group) (POR = 2.71, 95% CI: 1.14–6.63, *p* = 0.03). No other age group showed a significant difference relative to the reference group. Notably, there was no statistical association between the other variables and HPV infection in the multivariate model (Table 2). Factors such as vaccination status and type of sexual partner were excluded from the analysis because of data skewness.

**TABLE 2:** Risk factors associated with human papillomavirus infection (bivariate and multivariate analysis).

Variable	Unadjusted			Adjusted		
	*p*-value	POR	95% CI	*p*-value	POR	95% CI
**Employment**	0.13	-	-	-	-	-
Employed (ref)	-	-	-	-	-	-
Unemployed	0.31	0.79	0.50–1.24	0.71	0.91	0.56–1.48
Pensioner	0.07	0.38	0.12–1.01	0.10	0.21	0.03–1.20
**HIV status**	0.00	-	-	-	-	-
Negative (ref)	-	-		-	-	-
Positive	0.00	1.95	1.26–3.02	0.00	1.97	1.24–3.16
**Age**	0.13	-	-	-	-	-
18–28 (ref)	-	-	-	-	-	-
29–38	0.27	1.49	0.74–3.08	0.31	1.47	0.71–1.34
39–48	0.41	1.34	0.67–2.75	0.38	1.39	0.67–2.79
49–58	0.03	2.56	1.14–5.91	0.03	2.71	1.14–6.63
59+	0.85	0.92	0.36–2.28	0.33	1.86	0.51–6.49
**Age at first sexual intercourse (years)**	0.80	-	-	-	-	-
11–19 (ref)	-	-	-	-	-	-
16–20	0.86	0.94	0.48–1.89	-	-	-
21–25	0.82	1.10	0.47–2.61	-	-	-
25+	0.42	0.39	0.02–2.94	-	-	-
**Education**	0.08	-	-	-	-	-
Tertiary level (ref)	-	-	-	-	-	-
Primary and below	0.02	0.38	0.16–0.89	0.08	0.44	0.17–1.11
Secondary level	0.56	0.56	0.28–1.11	0.16	0.60	0.29–1.23
**Marital status**	0.98	-	-	-	-	-
Married (ref)	-	-	-	-	-	-
Never married	0.95	1.01	0.65–1.59	-	-	-
Widowed or divorced	0.88	0.94	0.41–2.07	-	-	-
**Income (South African Rand)**	0.79	-	-	-	-	-
Between R3000 and R10 000 (ref)	-	-	-	-	-	-
Greater than R10 000	0.75	0.85	0.30–2.40	-	-	-
Less than R3000	0.79	1.14	0.48–2.82	-	-	-
**Tobacco users**	0.90	-	-	-	-	-
Non-smoker (ref)	-	-	-	-	-	-
Smoker	0.90	1.08	0.27–3.87	-	-	-

POR, prevalence odds ratio; ref, reference; CI, confidence interval.

## Discussion

Infection with high-risk (hr) HPV is a global public health concern because of the greater risk of CC. Data on the prevalence and genotype distribution of HPV are scarce in the Limpopo province of South Africa. The current cross-sectional study determined the prevalence of cervical HPV infection and genotype distribution among 450 women living with and without HIV. Data on HPV prevalence and genotype distribution are crucial in evaluating the burden of viral infection in the studied population and assessing the effectiveness of prevention policies. To the best of our knowledge, this is the first study to investigate HPV infection among women living with and without HIV in Limpopo province.

Compared to a single-nested PCR approach, a double-nested reaction significantly enhanced the detection rate of HPV DNA in the study population. The study reports an overall prevalence of 32.7%, comprising both lr-HPV and hr-HPV infections. Subsequently, this finding aligns with findings from Tanzania (34.0%) and Central/Eastern Italy (33.0%).^20,24^ A slightly similar prevalence (28.5%) and much higher prevalences of 76.0% and 84.2% were reported in the Eastern Cape province of South Africa.^6,37,38^ In Africa and elsewhere, HPV prevalences also vary,^39,40^ and the contributing factors to the variability may include cervical cytology status, genotyping techniques,^41^ and study population variations.^42^ The high rates of HPV prevalence and ineffective cervical screening programmes in South Africa, where the rates of HIV are high, remain a significant public health concern.^37^ Implementation of effective preventative measures, such as HPV vaccinations, could benefit many in this population. Literature reports increased rates of HPV infection among WLWH compared to those without,^20^ which aligns with our study finding. This study further showed that WLWH were more susceptible to being infected with multiple infections and hr-HPV genotypes. Other studies have reported an increased risk of multiple infections, higher viral persistence and development of CC among WLWH.^21,43^ It is thought that HIV impacts the immune competence of the vaginal mucosa, thus promoting susceptibility to high-risk infections such as HPV.^44^ Moreover, there is a new paradigm in the history of HPV infection revealing that the loss of HPV detection likely reflects control of the immune system rather than complete viral clearance.^45^ Therefore, the high prevalence of HPV among WLWH compared to those living without HIV in the study population might be because of decreased immune competence.

In general, hr-HPV 45 (16.7%) emerged as the predominant genotype in our study population. This is similar to findings from KwaZulu-Natal province, where hr-HPV 45 (7.6%) was among the most commonly detected genotypes in a generalised population.^46^ A review study by Rikhotso and colleagues reported that hr-HPV 16, hr-HPV 18 and hr-HPV 35 are the predominant genotypes among South African women in the general population.^28^ The study’s findings suggest that the national HPV distribution patterns may not be an entirely reliable consensus for predicting provincial-level trends, necessitating the need for localised surveillance. Worldwide, hr-HPV 45 accounts for approximately 5% of all CC cases, with varying prevalences across regions in Eastern Asia (3%) and Africa (9%).^47,48^ The prevalence of hr-HPV 45 is thought to increase with the severity of cervical disease.^49^ Despite the cervical cytology status of the study participants being unknown, the high prevalence of hr-HPV 45 is concerning, mainly because only Cervarix and Gardasil-4 vaccines are currently in use in South Africa and do not directly target this genotype. However, evidence from a meta-analysis by Malagón and colleagues indicated that Cervarix may provide better cross-protection against hr-HPV 31, hr-HPV 33 and hr-HPV 45.^50^ Nonetheless, without adequate prevention or control measures, there is a potential for the continued transmission of hr-HPV 45, leading to increased risks for CC. Furthermore, besides hr-HPV 45, the distribution of the most frequently detected HPV genotypes varied between WLWH and women living without HIV. This finding is similar to reports from a study in Kigali, Rwanda.^51^ The application of NGS could have influenced the detection rate and types of HPV genotypes in this study. For example, hr-HPV 53, hr-HPV 56 and hr-HPV 66 not included in all three currently available HPV vaccines, were among the most common HPV genotypes detected. Therefore, including these genotypes in future HPV vaccines could enhance preventive benefits.

A study showed that the Gardasil-9 vaccine has been highly effective in preventing HPV infection and cancers, with efficacy rates of up to 100%.^52^ However, the vaccine is not yet available in South Africa, and over 50% of study participants in this study had at least one of the Gardasil-9 incorporated HPV genotypes. A previous sentinel surveillance study conducted in South Africa has also reported high rates of these genotypes.^27^ This study’s finding raises the importance of further engagement among policymakers to prioritise the Gardasil-9 vaccine rollout to reduce HPV transmission in South Africa. Moreover, the study’s findings could inform the vaccination campaign in Limpopo province, where data on HPV infection are limited.

The study also found a significant association between HIV positive status and HPV infection. This finding is consistent with a study conducted by Travill et al.^26^ Human immunodeficiency virus infection has a negative impact on the function of the immune system, thus promoting increased rates of HPV infection, viral persistence of hr-HPV and reactivation of latent HPV.^53,54^ A study conducted in the Western Cape province reported that WLWH were three times more likely to test positive for new HPV infection compared to their counterparts.^54^ Both HPV and HIV are STIs; therefore, educational campaigns in communities, including schools and healthcare centres, can help raise awareness about HPV infection and prevention, and contribute to reducing its burden. Moreover, the study’s findings showed a significant association between HPV infection and age, particularly among women aged 48 years – 59 years compared to those aged 18–28 years (reference group). Literature highlights that the younger age group tends to have transient HPV infections that they clear.^17^ Consequently, the South African CC guidelines recommend initiating screening on reaching 25 years of age at the earliest.^55^ The older women tend to have persistent infections that are likely to result in CC. These findings also support the vulnerability of older women, reinforcing the need for HPV targeted screening. The strengths of this study should be considered in the context of some limitations. This includes the lack of clinical information (such as cervical cytology, CD4 counts and antiretroviral therapy status), and a small sample size for HPV genotyping. Participants were enrolled from only four healthcare facilities in Limpopo province. Thus, the findings are an important indication but do not represent the general population in Limpopo province. Additional geographical inclusivity to understand the HPV genotype distribution on a broader scale would expand the scope and add more data to the current findings. Finally, the HIV status was self-reported and not verified through testing independently, which might affect the accuracy of the HIV-related data.

In conclusion, this study suggests a relatively high prevalence of HPV infection in the Limpopo province of South Africa, and that WLWH are more susceptible compared to those living without HIV. Therefore, prioritising WLWH for HPV screening and prevention strategies, such as vaccination, is recommended.
